# Socioeconomic Patterns of COVID-19 Clusters in Low-Incidence City, Hong Kong

**DOI:** 10.3201/eid2711.204840

**Published:** 2021-11

**Authors:** Gary K.K. Chung, Siu-Ming Chan, Yat-Hang Chan, Jean Woo, Hung Wong, Samuel Y. Wong, Eng Kiong Yeoh, Michael Marmot, Roger Y. Chung

**Affiliations:** The Chinese University of Hong Kong, Hong Kong, China (G.K.K. Chung, S.-M. Chan, Y.-H. Chan, J. Woo, H. Wong, S.Y. Wong, E.K. Yeoh, M. Marmot, R.Y. Chung);; City University of Hong Kong, Hong Kong (S.-M. Chan); University College London, London, UK (M. Marmot)

**Keywords:** COVID-19, coronavirus disease, SARS-CoV-2, severe acute respiratory syndrome coronavirus 2, viruses, respiratory infections, zoonoses, socioeconomic status, clustering, infection transmission, Hong Kong, China

## Abstract

Although coronavirus disease (COVID-19) outbreaks have been relatively well controlled in Hong Kong, containment remains challenging among socioeconomically disadvantaged persons. They are at higher risk for widespread COVID-19 transmission through sizable clustering, probably because of exposure to social settings in which existing mitigation policies had differential socioeconomic effects.

As coronavirus disease (COVID-19) continued to spread globally, studies of transmission mainly focused on clusters of >2 epidemiologically linked cases. Some governments, including those of New Zealand and Hong Kong, China, put specific focus on sizable infection clusters (i.e., clusters of >10 epidemiologically linked case-patients who are not all part of the same household) to detect widespread human-to-human COVID-19 infections with potentially greater numbers of successive transmission generations (*1*,*2*). These sizable infection clusters are closely linked to COVID-19 superspreading; as many as 7 superspreading events were related to the first few sizable infection clusters in Hong Kong (*3*). Given the widely observed higher COVID-19 incidence associated with socioeconomic disadvantages (*4*–*7*), determining whether the risk for sizable infection clustering is socioeconomically patterned is of public health significance. Such a pattern would imply not only higher risk for exposure to the virus but also increased risk of spreading the disease among socioeconomically disadvantaged communities.

Unlike many other parts of the world, Hong Kong has had a relatively low COVID-19 incidence, which made comprehensive contact tracing to identify sizable infection clusters possible and meaningful. In this study, we examined the association of socioeconomic position with sizable infection clustering in Hong Kong and explored the potential heterogeneity by case classification and different activity categories of clusters. For this study, we used data collected by the Centre for Health Protection (CHP), the Planning Department, and the Census and Statistics Department of the Hong Kong Government in compliance with the Declaration of Professional Ethics of the International Statistical Institute.

## The Study

We collected data on individual laboratory-confirmed cases from CHP (*1*) and a COVID-19 information website (*8*), which shows compiled information released by the CHP. During January 23–October 31, 2020, a total of 5,324 cases and 30 sizable infection clusters were identified (Appendix Table 1). We included 3,587 local cases with recognizable residential addresses in this study; 778 of those cases were linked to sizable infection clusters (Table 1).

**Table 1:** Characteristics of local coronavirus disease case-patients with a valid residential address, Hong Kong, 2020*

Characteristic	Total sample, N = 3,587	Area-level income poverty rate†			
		1st quartile	2nd quartile	3rd quartile	4th quartile
Mean age, y (SD)	47.92 (19.96)	44.20 (19.17)	47.66 (18.65)	49.93 (20.86)	46.63 (19.60)
Sex					
M	1,750 (48.8)	158 (51.6)	348 (47.4)	712 (50.6)	532 (46.6)
F	1,837 (51.2)	148 (48.4)	386 (52.6)	694 (49.4)	609 (53.4)
Sizable infection clustering					
Noncluster cases	2,809 (78.3)	275 (89.9)	617 (84.1)	1,033 (73.5)	884 (77.5)
Cluster cases‡	778 (21.7)	31 (10.1)	117 (15.9)	373 (26.5)	257 (22.5)
Living clusters	159 (4.4)	0 (0.0)	3 (0.4)	99 (7.0)	57 (5.0)
Working clusters	225 (6.3)	8 (2.6)	42 (5.7)	77 (5.5)	98 (8.6)
Dining clusters	248 (6.9)	15 (4.9)	35 (4.8)	137 (9.7)	61 (5.3)
Entertainment clusters	114 (3.2)	8 (2.6)	27 (3.7)	48 (3.4)	31 (2.7)
Others§	33 (0.9)	1 (0.3)	10 (1.4)	12 (0.9)	10 (0.9)
Case classification					
Infection source cases	1,455 (40.6)	133 (43.5)	317 (43.2)	528 (37.6)	477 (41.8)
Probable local cases	95 (2.6)	29 (9.5)	31 (4.2)	24 (1.7)	11 (1.0)
Local cases	1,360 (37.9)	104 (34.0)	286 (39.0)	504 (35.8)	466 (40.8)
Cases epidemiologically linked to infection source cases	2,132 (59.4)	173 (56.5)	417 (56.8)	878 (62.4)	664 (58.2)
Linked to probable local cases	62 (1.7)	12 (3.9)	20 (2.7)	22 (1.6)	8 (0.7)
Linked to local cases	2,070 (57.7)	161 (52.6)	397 (54.1)	856 (60.9)	656 (57.5)
Presence of symptoms					
Asymptomatic	590 (16.4)	44 (14.4)	89 (12.1)	262 (18.6)	195 (17.1)
Symptomatic	2,997 (83.6)	262 (85.6)	645 (87.9)	1144 (81.4)	946 (82.9)
Type of housing					
Public rental housing	1,479 (41.2)	6 (2.0)	243 (33.1)	591 (42.0)	639 (56.0)
Subsidized home ownership	409 (11.4)	6 (2.0)	137 (18.7)	171 (12.2)	95 (8.3)
Private housing	1,377 (38.4)	261 (85.3)	307 (41.8)	469 (33.4)	340 (29.8)
Residential care homes	116 (3.2)	3 (1.0)	6 (0.8)	86 (6.1)	21 (1.8)
Other	206 (5.7)	30 (9.8)	41 (5.6)	89 (6.3)	46 (4.0)
Area-level population density#					
1st quartile	409 (11.4)	82 (26.8)	165 (22.5)	102 (7.3)	60 (5.3)
2nd quartile	752 (21.0)	91 (29.7)	177 (24.1)	275 (19.6)	209 (18.3)
3rd quartile	888 (24.8)	55 (18.0)	200 (27.2)	310 (22.0)	323 (28.3)
4th quartile	1,538 (42.9)	78 (25.5)	192 (26.2)	719 (51.1)	549 (48.1)

*Values are no. (%) except as indicated. We used data current to October 31, 2020. †The 1st quartile is the wealthiest group and 4th quartile the poorest group. ‡The number of cluster cases differed from the sum of cluster cases across cluster types because one case was involved in both dining and working clusters. §Traveling, religious, grocery shopping activities. #The 1st quartile is lowest population density and 4th quartile the highest density.

We assigned as the dependent variable whether a case belonged to a sizable infection cluster. These sizable infection cluster cases included the earliest identified unlinked source cases and their subsequent epidemiologically linked cases. We categorized these clusters as living, working, dining, or entertainment (>100 cases each) on the basis of the type of activities most closely associated with the venues at which the source cases of each corresponding cluster were identified.

We adopted self-reported residential addresses of the confirmed case-patients (*8*) to generate 2 proxy socioeconomic measures (Appendix). First, we calculated the area-level income poverty rates as the proportion of households living at <50% of the median monthly household income for the corresponding household size in each of the 154 small-area Tertiary Planning Units (*9*); we then grouped these rates into quartiles. Second, we categorized the individual-level housing type into public rental housing, subsidized home ownership, private housing, residential care homes, and others (e.g., villages, industrial and commercial buildings, and staff quarters).

Results of multilevel binary logistic regression with random intercepts at area level showed that case-patients living in the wealthiest areas (i.e., 1st quartile) were 65% less likely to be cases in sizable infection clusters (adjusted OR [aOR] 0.35, 95% CI 0.19–0.65) than those living in the poorest areas (i.e., 4th quartile), after adjusting for confounding factors (Table 2). Area-level socioeconomic patterns of sizable clustering were more apparent among case-patients epidemiologically linked to previously confirmed cases (aOR 0.34, 95% CI 0.18–0.66) than among unlinked source cases (aOR 0.61, 95% CI 0.19–1.97). Such patterns were more pronounced for those in living and working clusters than in dining and entertainment clusters. At the individual level, persons living in residential care homes tended to be part of living-related sizable infection clusters. We observed stark variations in the effect of private housing across cluster categories; case-patients living in private housing had lower odds of being in working clusters (aOR 0.66, 95% CI .45–0.96) but increased odds of being in entertainment clusters (aOR 3.20, 95% CI 1.79–5.72) compared with case-patients living in public rental housing. 

**Table 2:** Associations of poverty rate and housing type with sizable coronavirus disease clustering, Hong Kong, 2020*

Category	aOR (95% CI)†							
	Total samples‡	Case classification			Specific activity categories‡			
		Unlinked‡	Linked‡		Living§	Working§	Dining§	Entertainment§
Area-level income poverty rate¶								
4th quartile	Referent	Referent	Referent		Referent	Referent	Referent	Referent
3rd quartile	0.89 (0.58–1.37)	1.27 (0.73–2.19)	0.81 (0.50–1.29)		0.61 (0.14–2.71)	0.83 (0.46–1.49)	1.00 (0.55–1.81)	1.13 (0.54–2.34)
2nd quartile	0.67 (0.42–1.06)	0.85 (0.42–1.74)	0.64 (0.39–1.07)		0.18 (0.02–1.52)	0.70 (0.37–1.34)	0.82 (0.43–1.56)	0.92 (0.42–2.06)
1st quartile	0.35 (0.19–0.65)	0.61 (0.19–1.97)	0.34 (0.18–0.66)		NA#	0.33 (0.13–0.87)	0.85 (0.37–1.92)	0.47 (0.16–1.35)
Individual-level housing type								
Public rental housing	Referent	Referent	Referent		Referent	Referent	Referent	Referent
Subsidized home ownership	0.97 (0.72–1.31)	1.26 (0.63–2.52)	0.99 (0.69–1.40)		1.22 (0.33–4.49)	0.72 (0.44–1.17)	1.06 (0.70–1.59)	1.27 (0.53–3.06)
Private housing	0.99 (0.77–1.26)	0.86 (0.49–1.51)	1.05 (0.79–1.39)		1.12 (0.46–2.72)	0.66 (0.45–0.96)	0.90 (0.62–1.32)	3.20 (1.79–5.72)
Residential care homes	27.20 (14.16–52.26)	4.69 (0.88–24.97)	22.35 (10.00–49.96)		720.16 (224.14–2,313.84)	NA**	NA#	NA#
Other	0.82 (0.51–1.33)	0.70 (0.22–2.27)	0.84 (0.49–1.46)		3.34 (0.87–12.81)	1.03 (0.53–1.99)	0.27 (0.09–0.82)	1.90 (0.71–5.09)

*Clustering for these data refers to >10 epidemiologically linked case-patients who are not all part of the same household, grouped by case classification and activity categories of clusters. aOR, adjusted odds ratio; NA, not available; Ref, reference. †Variables in the regression model were age (continuous), sex, presence of symptoms, type of housing, area-level income poverty rate (by quartiles), and area-level population density (by quartiles). ‡With reference to confirmed cases who were not classified into any sizable infection clusters. §With reference to confirmed cases who were not classified into the corresponding activity category of sizable infection clusters. ¶The 1st quartile is the wealthiest group and 4th quartile the poorest group. #No living cluster cases in the 1st quartile of area-level income poverty rate. **No cases living in residential homes for respective types of clusters.

## Conclusions

This study showed that socioeconomic disadvantage was associated with a wider COVID-19 transmission in the form of sizable infection clustering regardless of epidemic waves (Appendix Table 2); we observed a stronger socioeconomic pattern in clusters of more essential activities (i.e., living and working) than in clusters of less essential activities (i.e., dining and entertainment). The more apparent socioeconomic pattern of sizable COVID-19 clustering among epidemiologically linked cases suggested that the socioeconomically disadvantaged were not necessarily more prone to contracting the disease from random infection sources but that, once they contracted the disease, their communities were at higher risk for wide transmission of disease.

The stringent social distancing policies imposed by the Hong Kong government seriously disrupted social activities and confined residents to their own homes or local communities. The socioeconomically disadvantaged are particularly likely to be infected if they live in small, overcrowded apartments with poorer ventilation (*10*,*11*). Residential-care homes constituted 6 of 7 living-related infection clusters; these care homes tend to be located in socioeconomically disadvantaged areas, and sizable infection clusters involving care homes started to form when community outbreaks of local transmission became severe in early July 2020 (*1*,*12*). This observation implies that residential care home clusters are usually not only sporadic but also possibly concomitant with an outbreak in the disadvantaged community (*13*).

Work arrangement is another major COVID-19 containment measure with differential socioeconomic impacts. Despite advocacy for the work-from-home arrangement, the socioeconomically disadvantaged often could hardly benefit from this option (*5*). These persons also tend to work in occupations demanding longer hours and more intense social interactions and rely heavily on public transport, which inevitably increased their risk of having contact with infected persons and subsequently spreading the disease within their community. Moreover, the lack of financial subsidies to confirmed case-patients before late November 2020 may have kept these workers or the self-employed, who had no paid sick leave, from opting for necessary COVID-19 testing, thereby hampering early transmission containment. Altogether, we were not surprised to see several sizable infection clusters in the construction, transport, and direct-selling industries in Hong Kong.

Our results shed light on the pervasive social inequalities deeply entrenched in society. The socioeconomically disadvantaged have limited resources and opportunities to overcome structural constraints of the social environment (*14*) and are the ones hardest hit in emergencies or adverse events. The wealthier groups are at risk for infection through entertainment activities, given the propensity for widespread dispersion and difficulty in COVID-19 containment in these settings (*15*). Infection control may thus work better for the wealthier groups through restriction of entertainment activities.

A limitation of this study lies in the potential residual confounding as a result of the limited information the CHP released on the confirmed cases. In addition, case-patients who experienced symptoms after COVID-19 diagnosis may have been misclassified as asymptomatic. Moreover, we categorized the sizable infection clusters by social activities; therefore, infected case-patients epidemiologically linked to the source of one cluster were classified into the same activity category of the cluster regardless of their involvement with the specific activities.

In summary, despite relatively low COVID-19 incidence in Hong Kong, transmission containment among socioeconomically disadvantaged persons and communities remains challenging. Consideration of social inequalities is crucial to deploying equitable containment and exit strategies.

Appendix Additional information about socioeconomic patterns of coronavirus disease clusters, Hong Kong.
