# Supplementary material for: Socioeconomic Patterns of COVID-19 Clusters in Low-Incidence City, Hong Kong
Source: Emerg Infect Dis. 2021 Nov;27(11):2874–7. doi: 10.3201/eid2711.204840 (PMC8544972; doi:10.3201/eid2711.204840)
Supplement: Appendix — Additional information about socioeconomic patterns of coronavirus disease clusters, Hong Kong. [file 20-4840-Techapp-s1.pdf]

Article DOI: https://doi.org/10.3201/eid2711.204840

Socioeconomic Patterns of COVID-19 Clusters in Low-Incidence City, Hong Kong

Appendix

Measurement of Socioeconomic Positions

Self-reported residential addresses of the confirmed cases were adopted to generate proxy measures of their socioeconomic positions, and we derived 2 socioeconomic indicators (i.e., area-level income poverty rate and individual-level housing type).

For the area-level income poverty rate, we first classified the 4,086 cases into 154 large tertiary planning units (TPUs) demarcated by the Planning Department of Hong Kong for the Territory of Hong Kong (*1*). Then, based on the data of 2016 by-census from the Census and Statistics Department of Hong Kong, the median monthly household income with respect to household sizes were obtained (*1*). Hence, we estimated the number of households living with less than half of the corresponding median monthly household income by different household sizes in each TPU. By multiplying the estimated number of households by household sizes, we calculated the total number of persons living under income poverty in each TPU, and then divided it by the total population in the corresponding TPUs. Finally, we applied the resultant proportions (i.e., area-level income poverty rates) to all cases according to the corresponding TPUs of their residential addresses. The area-level income poverty rates in TPUs were grouped into quartiles for analysis.

As for the individual-level housing type, we categorized 5 groups of common housing type in Hong Kong based on their self-reported residential addresses, namely public rental housing, subsidized home ownership, private housing, residential care homes, and others (e.g., villages, industrial and commercial buildings, and staff quarters). Housing type was used to represent one's socioeconomic position because public rental housing and public subsidized housing have been offered to provide affordable housing for low-income households who meet stringent means-test of income and net asset, whereas those living in private housing tend to be wealthier given the great housing affordability stress in Hong Kong (*2*).

References

1. Census and Statistics Department. 2016 Population by-census: statistics by large Tertiary Planning Unit group. 2017 [cited 2021 Feb 22]. https://www.bycensus2016.gov.hk/en/bc-dp-tpu.html

2. Chung RY, Chung GK, Gordon D, Mak JK, Zhang LF, Chan D, et al. Housing affordability effects on physical and mental health: household survey in a population with the world's greatest housing affordability stress. J Epidemiol Community Health. 2020;74:164–72. PubMed https://doi.org/10.1136/jech-2019-212286

Appendix Table 1. Summary of coronavirus disease cases involved in sizeable infection clusters, Hong Kong*

Activity	Sizeable-infection clusters	No. confirmed cases
Living	Kong Tai Care for the Aged Centre	46
Living	Ming Chuen House of Shui Chuen O Estate	12
Living	The Salvation Army Lung Hang Residence for Senior Citizens	15
Living	Cornwall Elderly's Home (Golden Branch), Tuen Mun	40
Living	Sham Shui Po King Fok Nursing Home	14
Living	Hong Chi Lei Muk Shue Hostel	24
Living	The Home of Treasure	20
Working	Kerry Logistics / Luk Chuen House	12
Working	King Shing (Leung's) General Contractors Ltd./ Millennium City 3	32
Working	Star Global Direct Sales	61
Working	Sheung Shui Slaughter House	24
Working	Construction site at Kong Nga Po, Man Kam To	23
Working	Kwai Tsing Container Terminals	77
Working	Transport City Building, Tai Wai	17
Dining	Hotpot dinner gathering at Kwun Tong	13
Dining	Bun Kee / Sun Fat / Kin Wing	46
Dining	Tsz Wan Shan Shopping Centre Green River	38
Dining	Tsz Wan Shan Shopping Centre Windsor	26
Dining	Grand Plaza Taoheung	42
Dining	Tuen Mun Central Square Fulum Restaurant	44
Dining	Metroplaza Chiuchow Garden	20
Dining	Tsz Wan Shan Shopping Centre Deluxe Cuisine	32
Dining	Metropark Hotel Mongkok Victoria Harbour Restaurant	15
Entertainment	Bar and band cluster	103
Entertainment	Wedding party at Lantau Island	15
Entertainment	China Secret/Holly Mansion	15
Others	Diamond Princess Cruise Ship	11
Others	Fook Wai Ching Che in Maylun Apartments in North Point	19
Others	Travel tour to Egypt / Heng Tai House of Fu Heng Estate	10
Others	Hung Hom Market	13

*A sizable-infection cluster is defined as $\geq$10 epidemiologically linked case-patients who are not all part of the same household.

Appendix Table 2. Associations of area-level income poverty rate and individual-level housing type with sizable infection clusters by coronavirus disease epidemic period, Hong Kong*

			Epidemic period			
	Total sample, N = 3,587		Before April 30, 2020, N = 369†		After May 1, 2020, N = 3,218†	
Category	aOR (95% CI)‡	p value	aOR (95% CI)‡	p value	aOR (95% CI)‡	p value
Area-level income poverty rate						
4th quartile	Ref		Ref		Ref	
3rd quartile	0.89 (0.58–1.37)	0.591	0.98 (0.37–2.62)	0.974	0.75 (0.48–1.18)	0.217
2nd quartile	0.67 (0.42–1.06)	0.089	0.58 (0.21–1.61)	0.296	0.55 (0.33–0.90)	0.018
1st quartile	0.35 (0.19–0.65)	<0.001	0.24 (0.07–0.79)	0.019	0.31 (0.15–0.63)	0.001
Types of housing						
Public rental housing	Ref		Ref		Ref	
Subsidized home ownership	0.97 (0.72–1.31)	0.834	0.50 (0.17–1.47)	0.207	1.00 (0.73–1.37)	0.985
Private housing	0.99 (0.77–1.26)	0.905	0.76 (0.34–1.70)	0.510	0.84 (0.64–1.10)	0.211
Residential care homes	27.20 (14.16–52.26)	<0.001	NA§		25.94 (13.36–50.34)	<0.001
Others	0.82 (0.51–1.33)	0.421	0.80 (0.18–3.48)	0.764	0.82 (0.48–1.39)	0.450

*aOR, adjusted odds ratio; NA, not applicable; Ref, reference.

†With reference to confirmed cases who were not classified into any sizable infection clusters.

‡Variables in the regression model include age (continuous), sex, presence of symptoms at diagnosis, type of housing, area-level income poverty rate (by quartiles, with 1st the wealthiest and 4th the poorest), and area-level population density (by quartiles).

§No confirmed cases were identified in residential care homes before April 30, 2020.
